# Calomel and Tartar Emetic in the Army

**Published:** 1863-08

**Authors:** 


### SELECTED.

### CALOMEL AND TARTAR EMETIC IN THE ARMY.

The recent order of the Surgeon-General striking calomel and tartar emetic from the Supply List, has naturally excited a large amount of discussion and criticism in popular and professional circles. Quacks of every shade and complexion, from the infinitesimalist to the humblest dealer in roots and herbs, are exceedingly jubilant and profusely congratulatory. The old-school practitioner, who has to grapple daily with disease in all its multiplied forms, and solve the abstruse questions in therapeutics by actual experimentation, and who has come to give his faith to calomel and tartar emetic as his unfailing weapons in affections of great severity, fancies that a deadly blow has been aimed at his long tried and faithful allies. But students in the school of modern pathology and physiology look on with indifference, feeling that they have little or nothing at stake in the issue.

Thus far in the discussion of this order two questions have been raised :—1st, The propriety of the order, and, 2d, The propriety of removing from the Supply Table the articles referred to. The consideration of these two propositions evidently covers the whole ground. Let us examine them in detail.

It will not be denied that it is the duty of the Surgeon-General to regulate the Medical Supply Table. Annually, or oftener if necessary, the list of remedial agents to be employed by the Medical Staff must be revised, and such remedies added or stricken from it as the service may seem to demand. And the aggregate quantity to be used within a given time for a given force is also prescribed. No one questions the necessity or propriety of this revision of the Supply Table. It has never yet been alleged that in the discharge of this duty the Surgeon-General was impertinently interfering with the practice of the Medical Staff. Nor until now did it ever occur to any one that by this act the Surgeon-General reflected upon the professional qualifications of the surgeons of the army. On the contrary, the army surgeon has always been gratified at the revision of the table, for it seldom happened that new and important remedies were not added, and old and obsolete compounds stricken off.

The reason given in the order for striking calomel from the Supply Table is its abuse by military surgeons. The Surgeon-General states that he is officially informed that not only has profuse salivation been produced in innumerable cases, but that mercurial gangrene is of not infrequent occurrence. Finding it impossible to properly restrict the use of this powerful agent, he has ordered it stricken from the list of remedies furnished by the Department. He adds that he issues such an order with the more confidence, "as modern pathology has proved the impropriety of the use of mercury in very many of those diseases in which it was formerly unhesitatingly administered." That the Surgeon-General has presented sufficient reasons for his orders no one not stubbornly wedded to antiquated ideas can deny. The correctness of his information in regard to the lamentable consequences following the abuse of calomel no one will doubt who has visited many military hospitals, and inquired particularly as to the practice. A certain class of physicians almost invariably employ calomel, and always administer it in cases of doubtful diagnosis; and they are not satisfied unless they produce its constitutional effects, believing that it is only under such circumstances that it is effectual. We have known of its exhibition in military hospitals to salivation in chronic diarrhœa, Bright's disease, chronic rheumatism, etc. But it may be said that in certain diseases it is acknowledged by all authorities to be useful, and is the army surgeon to be deprived of mercurials because certain persons abuse calomel? We answer, certainly not. There are still on the Supply Table several of the more eligible and useful preparations of mercury. There is the blue mass, mercury and chalk, bichloride, iodide, etc., all much more elegant than calomel, and far more likely to give the beneficial effects of mercury without the unfavorable results.

That modern pathology has very much limited the class of diseases to which mercurials have been considered especially applicable, is apparent on every page of our recent works on practical medicine and surgery. Within twenty years, from being one of the most frequently employed agents of the materia medica, it has come to take a very subordinate position. And it should be a cause of sincere congratulation with every practitioner that, with the advance of modern pathology, a remedy of so much power for evil, if injudiciously used, is gradually being supplanted, whether by a more correct knowledge of its therapeutical uses, or by more eligible and more intrinsically harmless agents. The same remark is true of tartar emetic. This article the Surgeon-General has also stricken from the Supply Table, "for the reason that diseases prevalent in the army may be treated as efficiently without tartar emetic as therewith."

After a careful review of this subject, with an extended observation among the military hospitals, and of inquiry among army surgeons, we are compelled to regard the order of the Surgeon-General as a judicious, and even a necessary measure. In the opinion of the best medical officers, in no other way could the evil have been successfully reached. Fully confirmed in this view, we must regard the harsh criticisms of the Surgeon-General by certain medical conservatives as exceedingly unwise and unjust. We believe that in this order, as in all his official acts, he has not only endeavored to advance the best interests of the army medical service, but equally to maintain the honor and dignity of the profession of which he is a distinguished member.—*Am. Med. Times.*

